# Effect of optimized thrombus aspiration on myocardial perfusion and prognosis in acute ST-segment elevation myocardial infarction patients with primary percutaneous coronary intervention

**DOI:** 10.3389/fcvm.2023.1249924

**Published:** 2023-10-04

**Authors:** Boning Xu, Chunxin Zhang, Wei Wei, Yun Zhan, Mingguo Yang, Yanjun Wang, Jiajian Zhao, Guiyang Lin, Wen-wen zhang, Xing Huo, Bin Shi, Ling Fan

**Affiliations:** ^1^ Cardiovascular Department, The Fifth Clinical College of China Medical University-Bengang General Hospital of China Resources Medical Group, Benxi, China; ^2^ Cath Lab, The Fifth Clinical College of China Medical University-Bengang General Hospital of China Resources Medical Group, Benxi, China

**Keywords:** optimized thrombus aspiration, ST-segment elevation myocardial infarction, microcirculation disturbance, myocardial perfusion, infarct-related artery

## Abstract

**Objective:**

To investigate the impact of optimized thrombus aspiration on myocardial perfusion, prognosis, and safety in patients with acute ST**-**segment elevation myocardial infarction (STEMI) undergoing primary percutaneous coronary intervention(primary PCI).

**Methods:**

A total of 129 patients with STEMI were randomly allocated into control group (Subgroup A and B) and experimental group(Subgroup C and D). Control group received percutaneous transluminal coronary angioplasty (PTCA),thrombus aspiration and primary PCI. Experimental group received optimized thrombus aspiration and primary PCI. The number of thrombus aspiration was less than 4 times in Subgroup A and C. The number of thrombus aspiration was performed more than 4 times in Subgroups B and D. The classification of thrombi extracted, the TIMI flow grade, the incidence of no-reflow and slow flow, cTFC, TPI and CK-MB at 12 h and 24 h after stenting, ST segment resolution of ECG after stenting, NT-proBNP, LVEFat 24 h, 30 days and 180 days after stenting were compared between groups. The incidence of intraoperative and postoperative bleeding complications, stroke events and major cardiovascular events (MACE) were recorded and compared between groups.

**Results:**

The classification of thrombi extracted in the experimental group was higher than that in the control group. The TIMI flow grade of the experimental group was better than the control group after thrombus aspiration. After stenting, the advantage still existed, but the difference was not statistically significant. On cTFC, the experimental group was lower than the control group, but the difference was not statistically significant; After stenting the experimental group was significantly lower than the control group. The CK-MB at 12 h and 24 h of the experimental group was lower than the control group. After thrombus aspiration the incidence of no-reflow in the experimental group was significantly lower than that in the control group; after stenting the incidence of no-reflow in the experimental group was still lower than the control group, but no statistically difference. After thrombus aspiration and stenting the incidence of slow flow in the experimental group were lower than that in the control group. After stenting, NT-proBNP at 24 h was lower in the experimental group than that in the control group, However, there was no statistical difference; after stenting, The NT-proBNP in the experimental group was lower than that in the control group at 30 days and 180 days. After stenting, LVEF of the experimental group was significantly higher than the control group at 24 h and 30 days; superiority remained after 180 days but no statistical difference. There was no statistical difference between two groups for intraoperative and postoperative bleeding complications, stroke events, and MACE events. In Subgroup analysis,there was no significant difference in the classification of thrombi extracted, TIMI flow grade, cTFC, CK-MB,NT-proBNP and LVEF between group C and D, but group A was better than group B. Analysis of variance showed that the optimal number of suction was 4–5 times.

**Conclusions:**

Optimized thrombus aspiration can significantly improve myocardial perfusion and short-term and medium-term prognosis of STEMI patients after PCI, and reduce the incidence of slow flow and no-reflow. The optimal suction times were 4–5 times. Traditional aspiration method with more aspiration times is harmful to cardiac prognosis. Thrombus aspiration does not increase the incidence of stroke events and is safe.

**Clinical Trial Registration**: identifier, ChiCTR2300073410.

## Foreword

Acute myocardial infarction (AMI) has the highest mortality rate in cardiovascular diseases. The key to the treatment of AMI is to rapidly open the infarct-related artery (IRA), complete revascularization, and achieve effective reperfusion of the approaching necrotic myocardium. There are two commonly used clinical treatment methods, one is drug thrombolytic therapy; The second is primary percutaneous coronary intervention (PCI). Primary PCI is the most effective method for the treatment of acute myocardial infarction due to its high recanalization rate, sufficient and durable IRA recanalization and few bleeding complications. One of the more challenging aspects of primary PCI is thrombosis. The TAPAS study, presented at the EuroPCR meeting in 2008, was the first large randomized controlled study to demonstrate that thrombus aspiration vs. PCI alone significantly improves myocardial perfusion and outcomes in patients with STEMI. The 1-year follow-up showed that compared with the PCI group, the total death rate and the reinfarction rate in the thrombectomy group were significantly reduced (1). However, The TASTE study, published in 2013 in the New England Journal of Medicine, raised questions about thrombus aspiration when it showed no additional benefit of manual thrombus aspiration over PCI alone in reducing 30-day mortality in patients with STEMI. The results of the TOTAL trial, presented in 2015 at the ACC, showed that routine use of a manual aspiration catheter did not reduce the risk of cardiovascular death, recurrent myocardial infarction, cardiogenic shock, or heart failure at 180 days (similar to the results of the TASTE trial) but did increase the risk of embolic stroke at 30 days (different from the results of the TASTE trial) (2, 3).Thrombus aspiration has been downgraded from class ⅡA to Class Ⅱb in the guidelines in 2014 and 2017 years (4, 5). However, thrombus aspiration is recommended for patients with heavy thrombus burden during primary PCI, who are prone to coronary microcirculation dysfunction such as slow flow and no reflow, which aggravate myocardial injury and increase the incidence of heart failure and mortality (6, 7). In order to further search for the optimal treatment, this study adopted a randomized, parallel and controlled clinical trial design, to investigate whether the optimized thrombus aspiration method can improve the TIMI flow in patients with STEMI after primary PCI, and influence of cTFC, myocardial infarction size (ST segment resolution, TPI, CK-MB), clinical prognosis(NT-proBNP, LVEF) and the differences in safety were corrected. To further clarify whether optimizing thrombus aspiration can improve myocardial perfusion and prognosis in patients with STEMI, so as to provide new life and new perspective for the “ancient” method of thrombus aspiration therapy.

## Methods

### Patients population

A total of 129 STEMI patients were selected in Bengang General Hospital from November 4, 2020 to January 25, 2022(males 101, females 28),the maximum age is 86, the minimum age is 34, the average age is(59.3 ± 10.7).

Inclusion criteria:(1) Patients with acute ST-segment elevation myocardial infarction within 12 h of onset according to the international common diagnostic criteria were enrolled. (2) Coronary angiography showed 100% occlusion of IRA,vessel diameter ≥2.Omm,no obvious tortuosity and calcification. (3) There was no contraindication of antiplatelet and anticoagulant therapy. (4) With a state of high thrombus burden should be aspirated. All enrolled subjects conform to four inclusion criteria.

The state of high thrombus burden: Coronary angiography showed long thrombus more than three times the diameter of the reference vessel; floating thrombus at the proximal end of the occluded vessel; the proximal end of the occluded vessel had >5 mm long strip thrombus; sudden complete occlusion of the proximal vessel without tapering; coronary artery occlusion-related vessels >4.0 mm;contrast agent was retained distal to the occluded vessel.

Exclusion criteria: (1) patients had undergone rescue PCI with intravenous thrombolysis; (2) history of intracranial hemorrhage; (3) ischemic stroke within 3 months; (4) suspected aortic dissection; (5) active bleeding or bleeding factors; (6) severe head and face injury within 3 months; (7) severe or poorly controlled hypertension; (8) severe trauma or major surgery or bleeding from vessels that could not be compressed within 3 weeks; (9) patients with malignant tumors; (10) complicated with severe liver and kidney dysfunction; (11) pregnant, lactating or at risk of becoming pregnant; (12)known allergies or contraindications to study drugs or contrast media; (13) patients had participated in other clinical trials at the same time; (14) patients after coronary artery bypass grafting; (15) Coronary angiography showed TIMI grade 3 blood flow in the culprit vessel without obvious signs of thrombosis. (16) patients with acute STEMI more than 12 h; (17) patients with acute non-STEMI;(18) The lesion was located in the left main coronary artery, the anterior trigeminal artery, the vessel was tortuous and calcified, the lesion was diffuse, and multiple stents were implanted more than 3 pieces; (19) blood pressure ≤90/60 mmHg;shock; Severe heart failure.

This study was approved and consented to participate by the Ethics Committee of China Resources Medical Group Bengang General Hospital (Approval No.:No.1,2020-010, Bengang General Hospital Ethical Review).This study has been registered in the Chinese Clinical Trial Registry (registration number: ChiCTR2300073410; Reg Date:10/07/2023).Informed consent was obtained from all subjects. This study was consented to publication.

### Study design

A randomized, parallel, controlled design was used in this study, the random number table method was used for randomization. For STEAMI patients who conform to four inclusion criteria and did not meet the exclusion criteria, They were randomly divided into the control group(Subgroup A + B)(males 49, females 15)(average age 60.7 ± 11.7years)and experimental group (Subgroup C + D), (males52,females13) (average age57.9 ± 9.3years). Subgroup A included 32 patients(males 24, females 8) (average age 58.6 ± 8.3years).Subgroup B included 32 patients (males 25, females 7) (average age 62.8 ± 7years).Subgroup C included 33 patients (males 26, females 7)(average age 59.7 ± 10.2years).Subgroup D included 32 patients (males 26, females 6)(average age56.1 ± 9.7years).All patients underwent primary PCI, A loading dose of 300 mg aspirin (Bayer HealthCare Manufacturing S.r.l.Batch number: 3J71004) and Clopidogrel (Sanofi-aventis groupe, Batch number:BA992)300–600 mg or Ticagrelor (Astrazeneca Pharmaceuticals LTD. Batch number:2301050) 180 mg was given before surgery. In the control group, PTCA was performed before thrombus aspiration after emergency coronary angiography guide wire passage, Then, primary PCI was performed. In the experimental group, thrombus aspiration was performed directly after the emergency coronary angiography guide wire was passed, primary PCI was performed after thrombus aspiration was completed. Thrombus aspiration was Less than or equal to 4 times in groups A and C; Thrombus aspiration was performed more than 4 times in groups B and D. In the case of slow flow and no reflow during operation, Sodium Nitroprusside(Hainan Puli Pharmaceutical Co. LTD. Batch number:120721000) and Tirofiban[Broad Pharma (China) Co., LTD.Batch number:220802]can be injected into the coronary artery.

### Indicators of observation

(1) TIMI flow grade and cTFC after thrombus aspiration and stenting,the incidence of no-reflow and slow reflow,frequency and dosage of drug intervention during stenting. (2) Evaluate the grade of classification of aspirated thrombus.Grading methods of thrombus: Grade 0:no thrombus visible to the naked eye;Grade 1: the total length of thrombus aspirated was less than 5 mm; Grade 2: total length of aspirated thrombus 5–10 mm; Grade 3: The total length of the aspirated thrombus was greater than 10 mm. (3)Evaluate the amplitude of ST-segment resolution on ECG at 2, 4, 6, 12 and 24 h after stenting;the peak values of serum TPI and CK-MB at 12 h and 24 h after stenting. (4) NT-proBNP and LVEF at 24 h, 30 days and 180 days after stenting. (5) Intraoperative and postoperative bleeding complications,stroke events and MACE events (death, reinfarction, target vessel revascularization) at 30 days and 180 days after stenting were recorded.

### Statistical analysis

SPSS26.0 software was used for data processing. Measurement data were expressed as $\bar{X}\pm s$, *T*-test, analysis of variance and LSD *post hoc* test were used for comparison between groups. Count data were expressed as percentages, Rank sum test was used for comparison between groups. Logistic stepwise regression model was used for multivariate correlation. The cumulative incidence of stroke events and MACE events was detected by Kaplan-Meier method. *P* < 0.05 was considered statistically significant.

## Results

**Figure 1:**
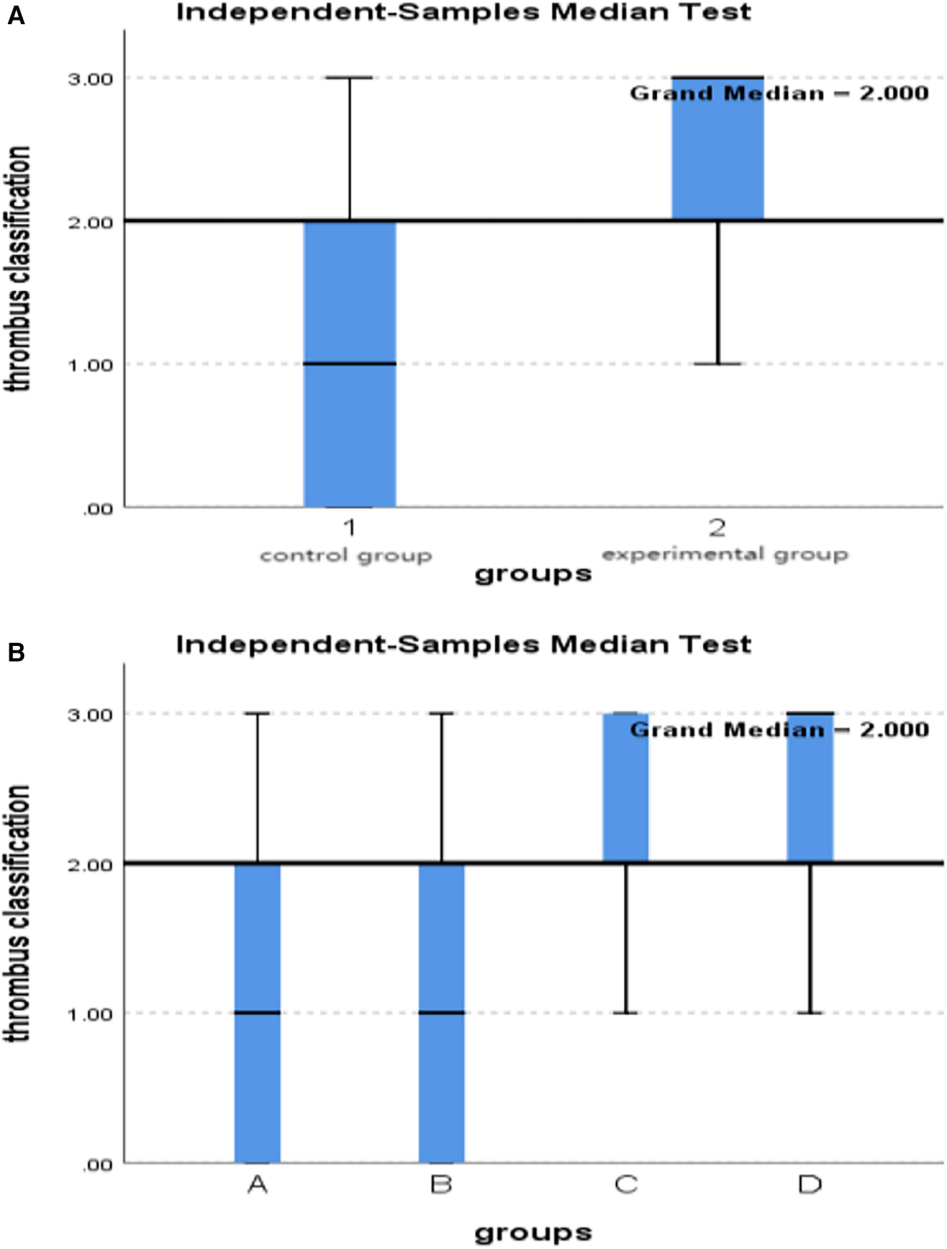
(**A**) Comparison of thrombus classification in two groups. (**B**) Comparison of thrombus classification among subgroups A, B, C and D.

**Figure 2:**
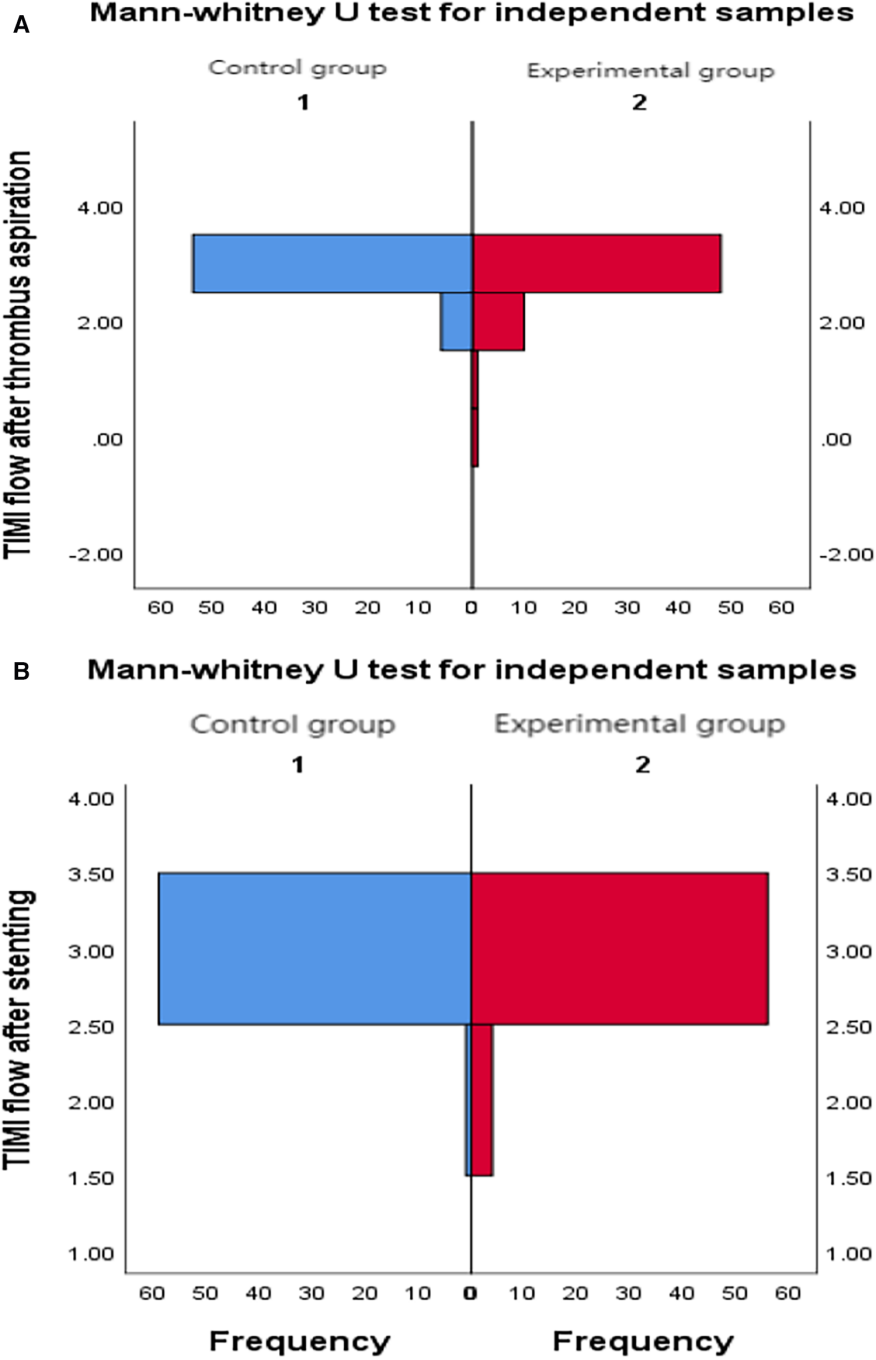
(**A**) Comparison of TIMI flow grade between two groups after thrombus aspiration. (**B**) Comparison of TIMI flow grade between two groups after stenting.

**Figure 3:**
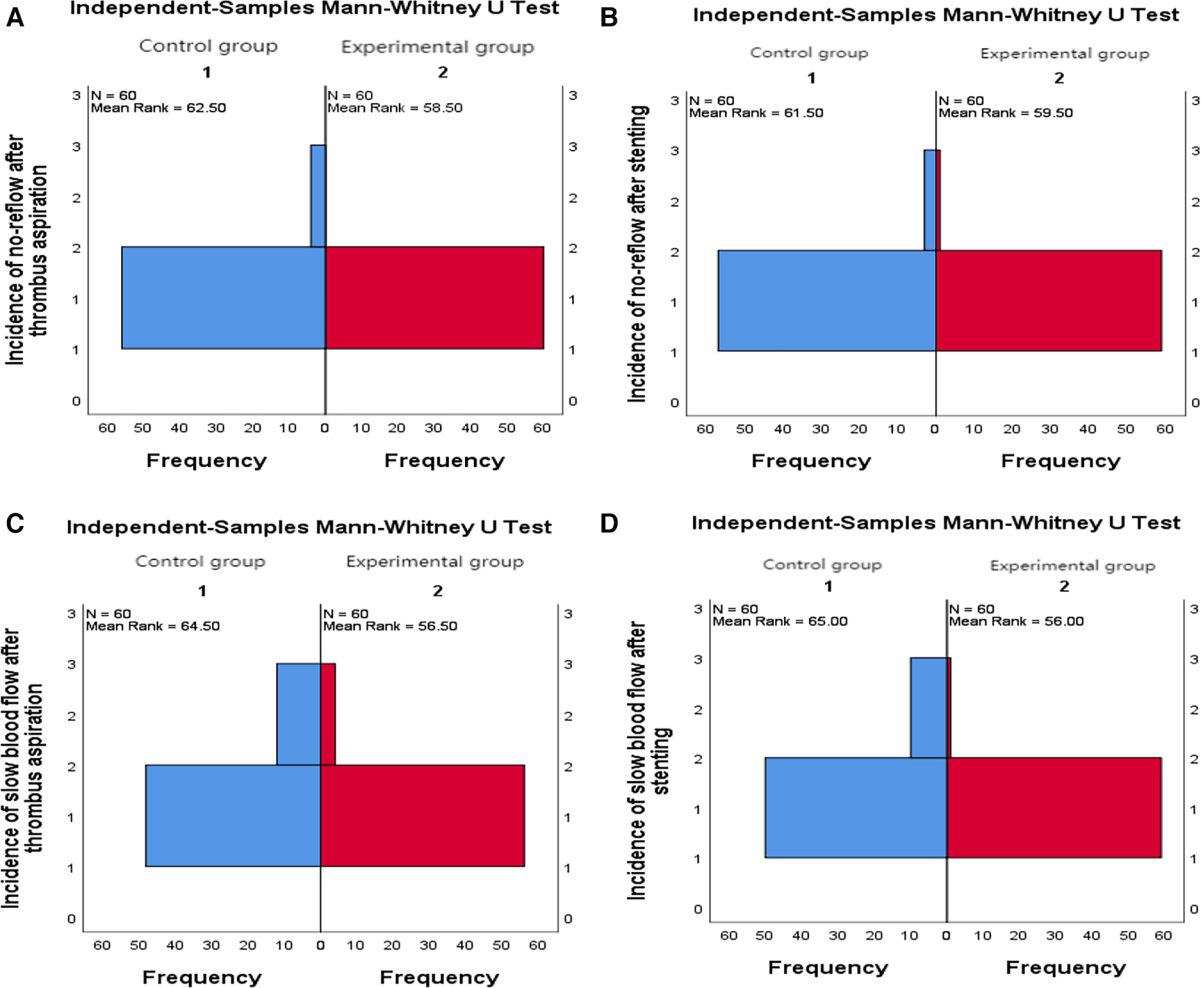
(**A**) Comparison of the incidence of no-reflow after thrombus aspiration between the two groups. (**B**) Comparison of the incidence of no-reflo after stenting between the two groups. (**C**) Comparison of the incidence of slow flow after thrombus aspiration between the two groups. (**D**) Comparison of the incidence of slow blood flow after stenting between the two groups.

**Figure 4:**
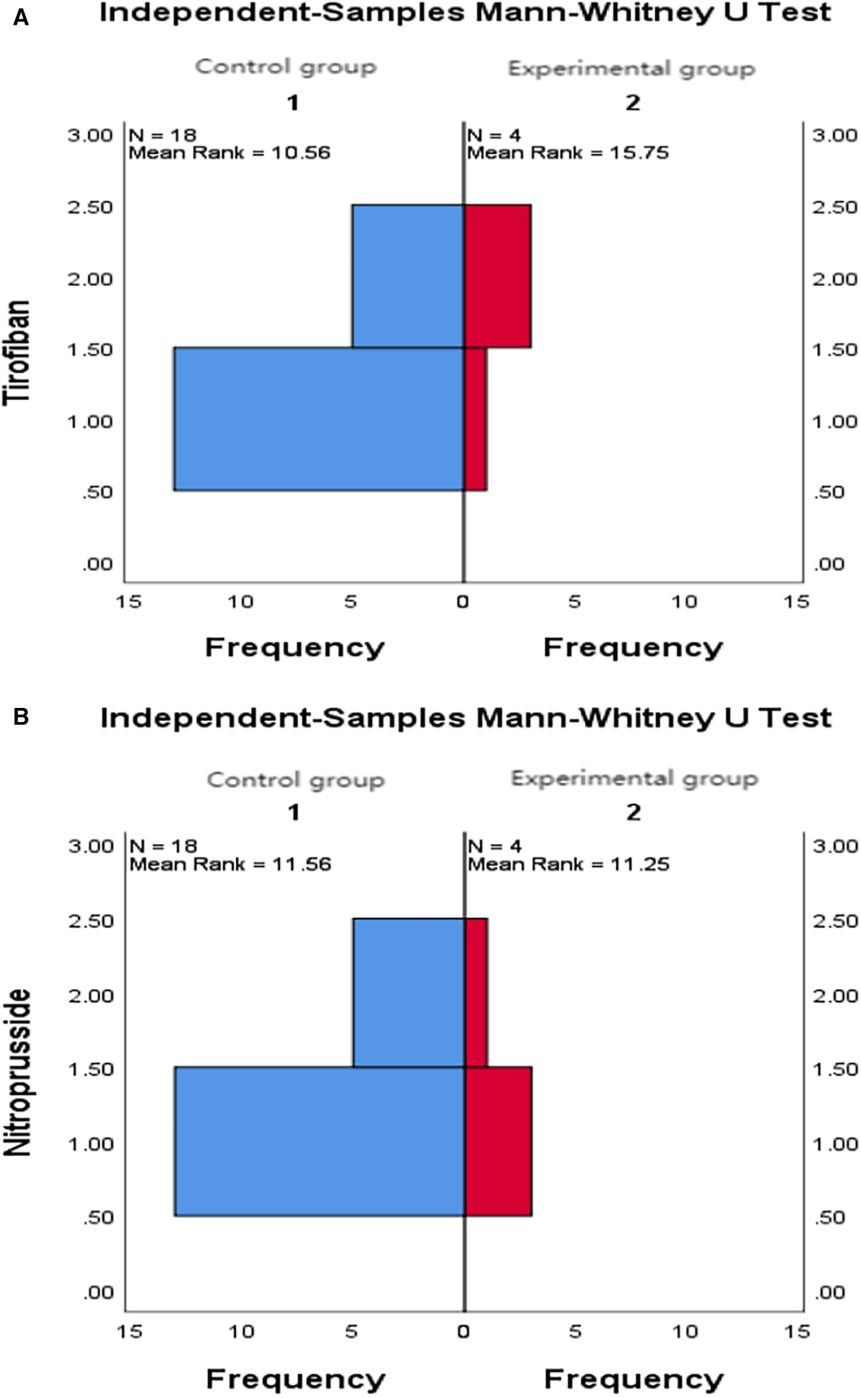
(**A**) Comparison of intraoperative tirofiban application between the two groups. (**B**) Comparison of intraoperative nitroprusside application between the two groups.

**Figure 5:**
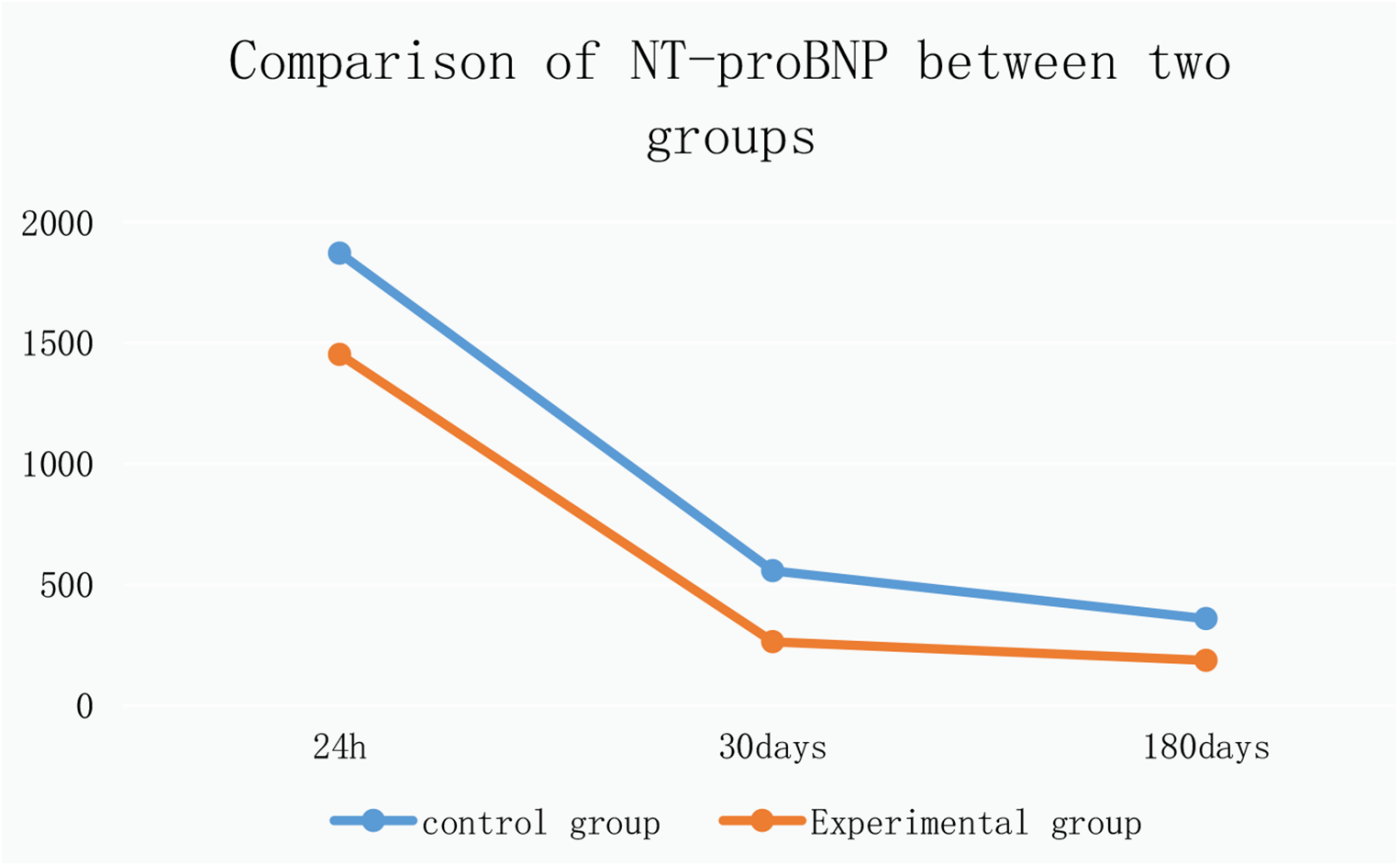
Comparison of NT-proBNP between control and experimental groups.

**Figure 6:**
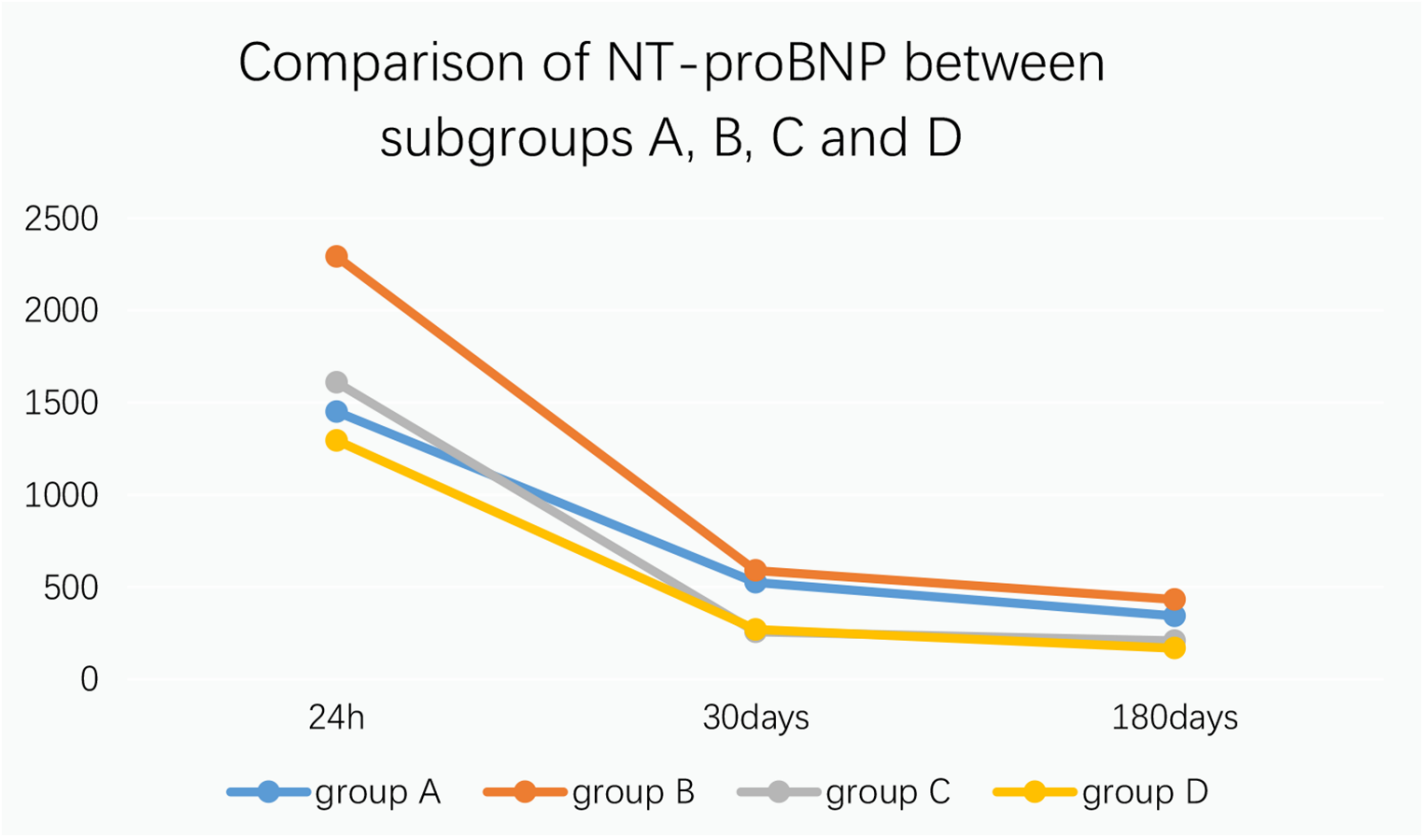
Comparison between NT-proBNP in subgroups A, B, C and D.

**Figure 7:**
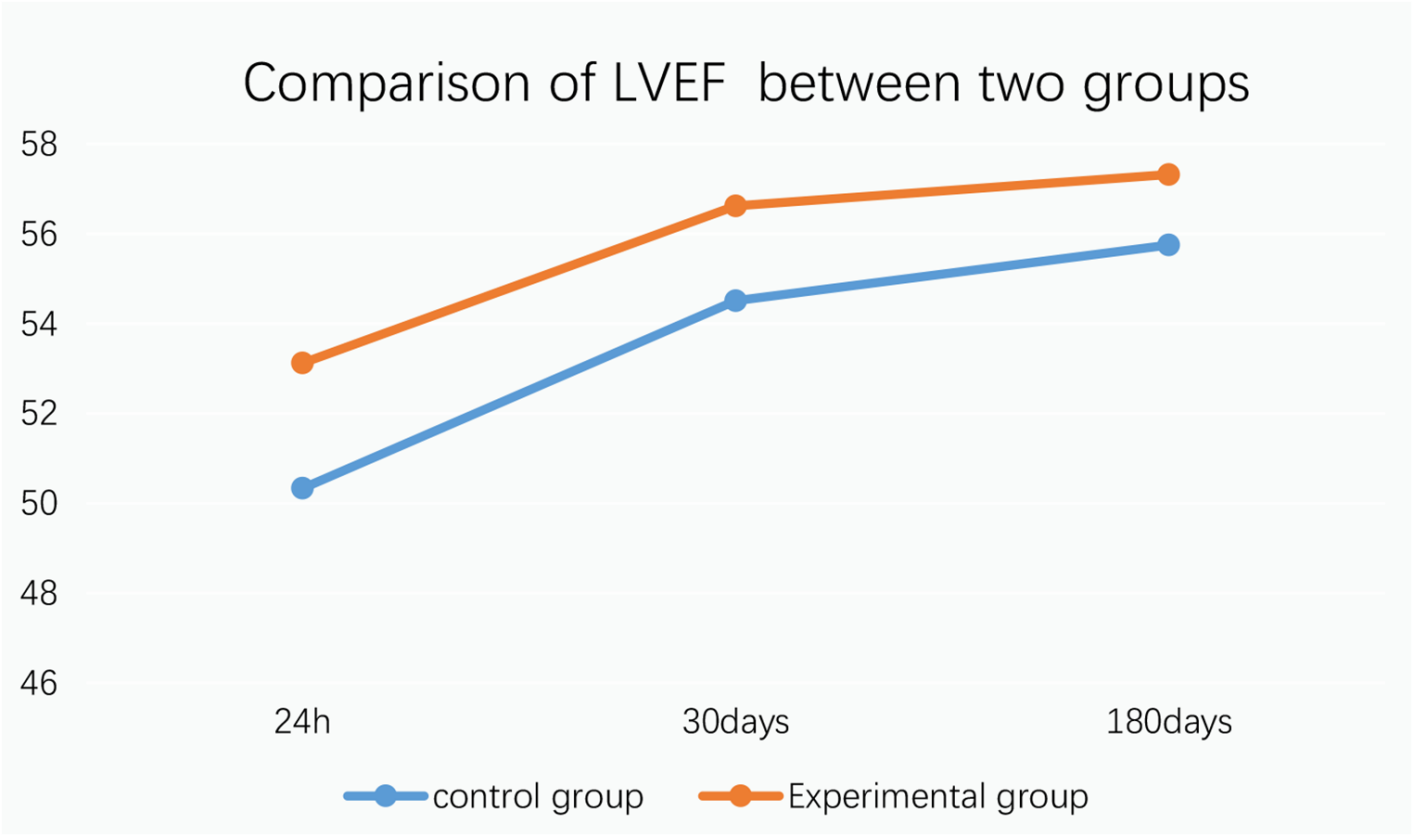
Comparison of LVEF between two groups.

**Figure 8:**
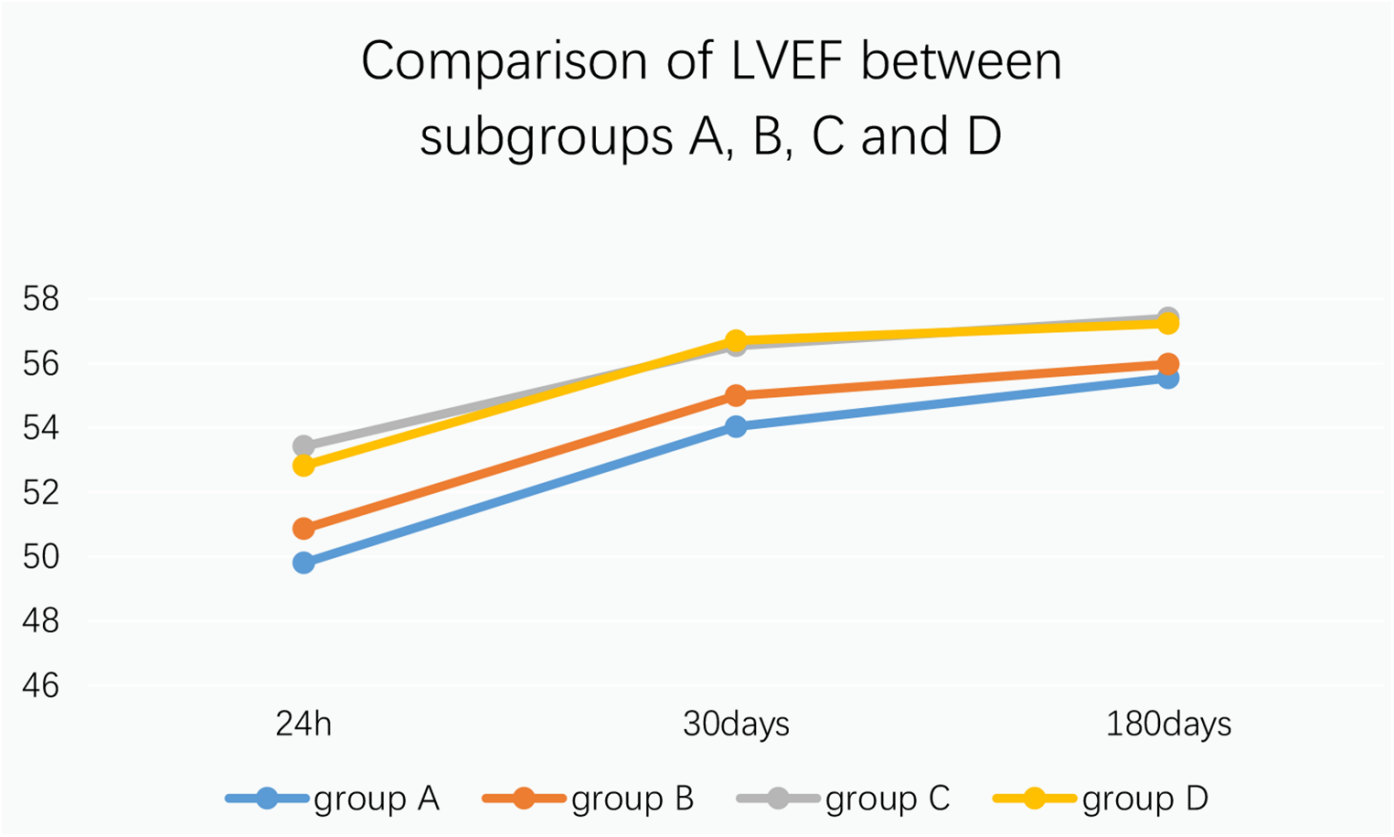
Comparison of LVEF between subgroups A, B, C and D.

**Figure 9:**
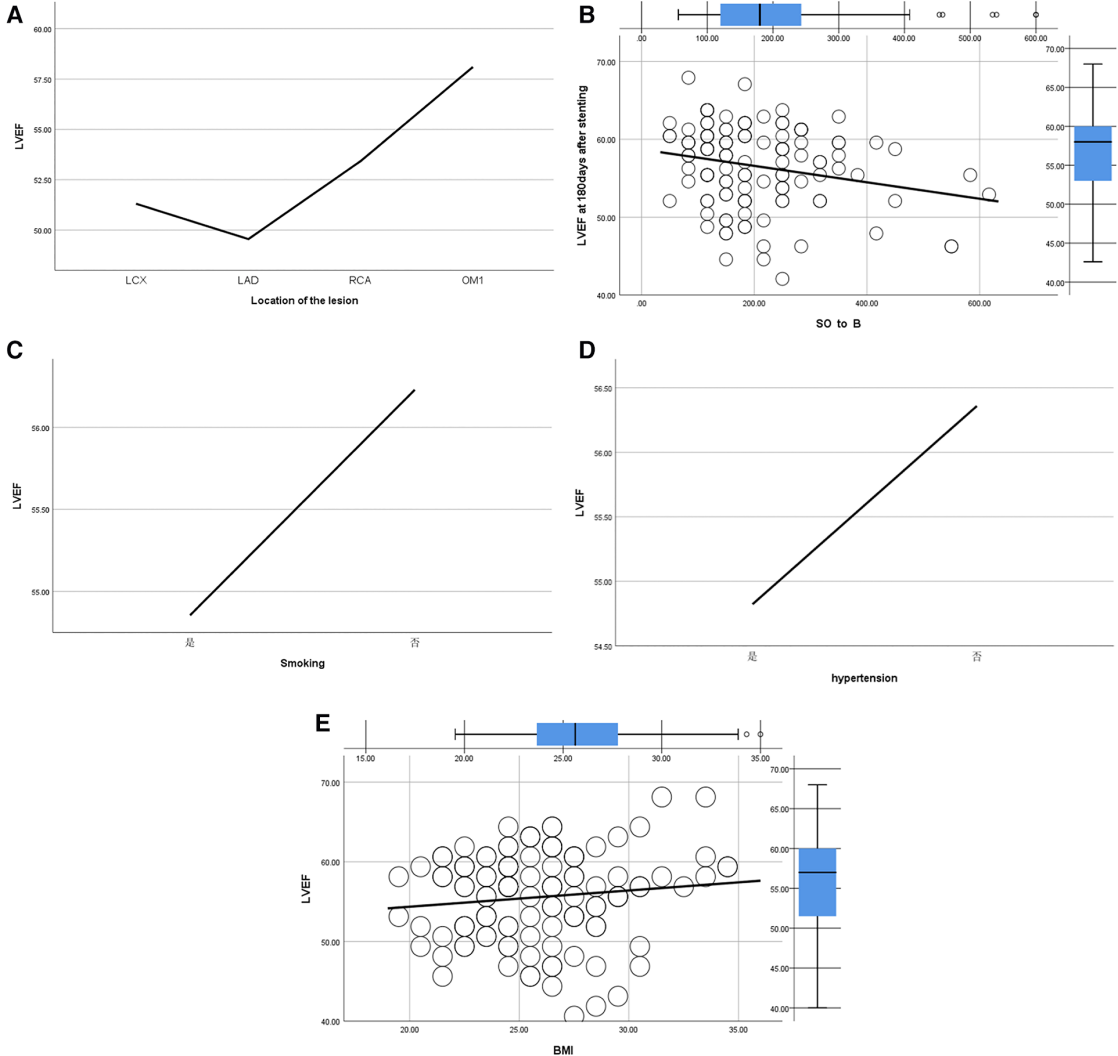
(**A**) Regression analysis of LVEF and location of the lesion. (**B**) Regression analysis of LVEF and SO_2_B. (**C**) Regression analysis of LVEF and smoking. (**D**) Regression analysis of LVEF and hypertension. (**E**) Regression analysis of LVEF and BMI.

1. A total of 129 patients were enrolled, a total of 9 cases were lost to follow-up (2 cases in group A, 2 cases in group B, 3 cases in group C and 2 cases in group D), The total loss rate of follow-up was 6.98%. A total of 120 cases completed the follow-up, including 60 cases in the control group (30 cases in group A, 30 cases in group B) and 60 cases in the experimental group (30 cases in group C, 30 cases in group D). The shortest follow-up time was 6 months and the longest was 21 months, The average follow-up time was (13.92 ± 4.18) months, and the median follow-up time was 14 months. The average times of thrombus aspiration in the four groups were (3.80 ± 0.48) times in group A, (6.63 ± 1.30) times in group B, (3.77 ± 0.43) times in group C and (6.60 ± 1.07) times in group D.
2. The measurement data of 120 patients were in accordance with normal distribution. There was no significant difference in age, gender, BMI, SO_2_B (Symptom onset to balloon time), D_2_B (Door to balloon time), lesion location,number of stents, concomitant diseases, risk factors, preoperative medication between the two groups by homogeneity of variance test (*P* > 0.05).
3. Comparison of thrombus aspiration classification between groups.
  3.1 Comparison of thrombus aspiration classification between control group and experimental group:After thrombus aspiration,the classification of thrombus in the experimental group was higher than that in the control group, and the difference was statistically significant (*P* = 0.008) (Table 1 and Figure 1A).
  3.2 Comparison of thrombus aspiration classification between subgroups A, B, C, and D:After thrombus aspiration,the classification of grade 2 and 3 thrombi extracted in groups C and D was higher than that in groups A and B, with statistically significant differences (*P* = 0.018) (Table 2 and Figure 1B).
4. Comparison of TIMI flow grade between-groups.
  4.1 Comparison of TIMI flow grade between control group and experimental group: The TIMI flow grade after thrombus aspiration in the experimental group was better than that in the control group (*P* = 0.03). After stenting, there was no significant difference in TIMI flow grade between the two groups (*P* = 0.17) (Table 3 and Figures 2A,B).
  4.2 Comparison of TIMI flow grade between subgroups A, B, C, and D: after thrombus aspiration, TIMI flow grade in group B was lower than that in groups A, C, and D (*P* = 0.009); After stenting, the TIMI flow grade in group B was lower than that in groups A and D (*P* = 0.03) (Table 4).
5. Comparison of cTFC between groups
  5.1 Comparison of cTFC between control group and experimental group: After thrombus aspiration, the cTFC of the experimental group was lower than that of the control group, and there was no significant difference between the two groups (*P* = 0.56). After stenting, the cTFC of the experimental group was lower than that of the control group, and the difference was statistically significant (*P* = 0.003) (Table 5).
  5.2 Comparison of cTFC between subgroups A, B, C, and D: The cTFC of group B was higher than that of group A, C and D after thrombus aspiration, and the difference was not statistically significant(*P* = 0.37). After stenting, the cTFC of group B was higher than that of groups A, C, and D (*P* = 0.001), and the CTFC of group C was better than that of groups A and D(*P* = 0.94) (Table 6).
6. There was no significant difference in ST segment resolution between the control group and the experimental group, and among the four subgroups A, B, C, and D (Tables 7, 8).
7. There was no significant difference in TPI at 12 h and 24 h after stenting between the control group and the experimental group, and among the four subgroups A, B, C and D (Tables 9, 10). (TPI was detected by chemiluminescence method, and some values exceeded the limit range, which may affect the results of statistical data.)
8. Comparison of CK-MB between groups
  8.1 Comparison of CK-MB between the control group and the experimental group: the peak value of CK-MB in the experimental group was lower than that in the control group at 12 h and 24 h after stenting,and the difference was statistically significant (*P* = 0.007, *P* = 0.016) (Table 11).
  8.2 Comparison of CK-MB values between subgroups A, B, C and D: CK-MB values in group B were significantly higher than those in groups A, C and D at 12 h and 24 h after operation (*P* = 0.006, *P* = 0.016) (Table 12).
9. Comparison of no-reflow and slow flow rates between groups
  9.1 Comparison of no-reflow and slow flow rates between the control group and the experimental group: The incidence of no-reflow in the experimental group was lower than that in the control group after thrombus aspiration (*P* = 0.04), and there was no significant difference between the two groups after stenting (*P* = 0.31).The incidence of slow flow in the experimental group was lower than that in the control group after thrombus aspiration and stenting (*P* = 0.03, *P* = 0.005) (Table 13 and Figures 3A–D).
  9.2 Comparison of no-reflow and slow flow rates values between subgroups A, B, C and D: The incidence of slow flow in group B was higher than that in group C and group D after stenting (*P* = 0.029) (Table 14).
10. Comparison of intraoperative drug intervention between the two groups: In order to obtain the same TIMI flow grade3 after stenting, the frequency of tirofiban in the experimental group was lower than that in the control group, and the difference was statistically significant (*P* = 0.002).The dosage of sodium nitroprusside in the experimental group was lower than that in the control group, and the difference was statistically significant (*P* = 0.001) (Table 15 and Figures 4A,B).
11. Comparison of NT-proBNP between groups
11.1 Comparison of NT-proBNP levels between the control group and the experimental group at 24 h, 30 days, and 180 days after stenting:The value of NT-proBNP in the experimental group was lower than that in the control group at 24 h after stenting, but the difference was not statistically significant (*P* = 0.156). The value of NT-proBNP in the experimental group was lower than that in the control group at 30 days and 180 days after stenting, and the difference was statistically significant (*P* = 0.002, *P* = 0.04) (Table 16 and Figure 5).
11.2 Comparison of NT-proBNP levels between subgroups A, B, C and D at 24 h, 30 days, and 180 days after stenting: The level of NT-proBNP in group B was significantly higher than that in group A and D at 24 h, 30 days and 180 days after stenting (*P* = 0.042). At 30 days after stenting, NT-proBNP in group B was significantly higher than that in group C and group D (*P* = 0.019), and NT-probnp in group A was significantly higher than that in group C and group D (*P* = 0.02). At 180 days after stenting, NT-proBNP in group B was significantly higher than that in group C and group D (*P* = 0.045) (Table 17 and Figure 6).
12. Comparison of LVEF between groups
12.1 Comparison of LVEF between the control group and the experimental group at 24 h, 30 days and 180 days after stenting: LVEF in the experimental group was significantly higher than that in the control group at 24 h and 30 days after stenting(*P* = 0.01, *P* = 0.04). LVEF in the experimental group was still higher than that in the control group at 180 days after stenting,but there was no significant difference between the two groups (*P* = 0.09) (Table 18 and Figure 7).
12.2 Comparison of LVEF between subgroups A, B, C and D at 24 h, 30 days and 180 days after stenting:Comparison of LVEF among subgroups A, B, C and D: LVEF in group A was lower than that in group C at 24 h after stenting(*P* = 0.03) (Table 19 and Figure 8).
13. Stepwise regression analysis of LVEF at 24 h, 30 days and 180 days after stenting, as shown in Table 20, LVEF at 24 h after stenting was highly correlated with the location of the lesion; LVEF at 30 days after stenting was highly correlated with lesion location, hypertension and smoking; LVEF at 180 days after stenting was highly correlated with SO_2_B, hypertension, smoking and BMI (Table 20 and Figures 9A–E).
14. By grading the suction times of 120 patients, the effects of different suction times on immediate myocardial blood flow, myocardial perfusion and cardiac function were analyzed, and the optimal suction times were analyzed. The influence of different suction times on myocardial perfusion and cardiac function prognosis was analyzed, and the optimal suction times was 4–5times (Table 21).
15. There was no significant difference in the cumulative incidence of intraoperative and postoperative bleeding events, stroke events and MACE events between the two groups by Kaplan-Meier analysis.

**Table 1:** Comparison of thrombus classification between the control group and the experimental group.

Thrombus classification Group	Grade 0 (%)	Grade 1 (%)	Grade 2 (%)	Grade 3 (%)	*H*	*P*
Control group	7.3	43.9	26.8	22.0		
Experimental group:	0	28.9	28.9	42.2	17.9	0.008

**Table 2:** Comparison of thrombus classification among subgroups A, B, C and D.

Thrombus classification	Grade 0 (%)	Grade 1 (%)	Grade 2 (%)	Grade 3 (%)	*H*	*P*
Group A	7.1	43.0	27.7	22.2		
Group B	7.5	44.8	25.9	21.8		
Group C	0	28.6	27.6	43.8		
Group D	0	29.2	30.2	40.6	11.9	0.018

**Table 3:** Comparison of TIMI flow grade between the control group and the experimental group.

TIMI flow grade Group		Grade 0 (%)	Grade 1 (%)	Grade 2 (%)	Grade 3 (%)	*H*	*P*
After thrombus aspiration	Control group	1.7	1.7	20.0	76.6		
	Experimental group	0	0	6.7	93.3	2104.0	0.03
After stenting	Control group	0	0	6.7	93.3		
	Experimental group	0	0	1.7	98.3	1890.0	0.17

**Table 4:** Comparison of TIMI flow grade among subgroups A, B, C and D.

TIMI flow grade Group		Grade 0 (%)	Grade 1 (%)	Grade 2 (%)	Grade 3 (%)	*H*	*P*
After thrombus aspiration	Group A	0	0	13.3	86.7		
	Group B	3.3	3.3	26.7	66.7^a,b,c^		
	Group C	0	0	6.7	93.3		
	Group D	0	0	6.7	93.3	11.61	0.009
After stenting	Group A	0	0	0	100		
	Group B	0	0	13.3	86.7^a,c^		
	Group C	0	0	3.3	96.7		
	Group D	0	0	0	100	8.90	0.03

The subgroup was analyzed in pairs.

^a^ Means group B compared with group A *P* < 0.05.

^b^ Means group B compared with group C *P* < 0.05.

^c^ Means group B compared with group D *P* < 0.05.

**Table 5:** Comparison of cTFC between control group and experimental group.

cTFC Group	After aspiration (30 fps)	After stenting (30 fps)
The control group	28.65 ± 17.09	21.03 ± 13.44
The experimental group was	26.95 ± 14.99	15.10 ± 6.63
*T*	0.58	3.07
*P*	0.56	0.003

**Table 6:** Comparison of cTFC in four subgroups A, B, C and D.

cTFC Group	After aspiration (30 fps)	After stenting (30 fps)
Group A	26.40 ± 13.81	16.77 ± 9.63
Group B	30.90 ± 19.83	25.30 ± 15.39^a,b,c^
Group C	24.30 ± 12.75	13.73 ± 6.39
Group D	29.60 ± 16.73	16.47 ± 6.68
*F*	1.05	7.26
*P*	0.37	0.001

Subgroups were analyzed by LSD method *post hoc* test.

^a^ Means group B compared with group A *P* < 0.05.

^b^ Means group B compared with group C *P* < 0.05.

^c^ Means group B compared with group D *P* < 0.05.

**Table 7:** Comparison of ST segment resolution after stenting between the control group and the experimental group.

The ST segment resolution after stenting	2 h (mv)	4 h (mv)	6 h (mv)	12 h (mv)	24 h (mv)
The control group	4.19 ± 2.28	4.15 ± 2.27	4.23 ± 2.28	4.19 ± 2.29	4.38 ± 2.21
Experimental group	4.09 ± 2.54	4.09 ± 2.61	4.09 ± 2.67	4.05 ± 2.72	4.14 ± 2.78
*T*	0.15	0.09	0.19	0.21	0.34
*P*	0.89	0.93	0.85	0.84	0.7

**Table 8:** Comparison of ST segment resolution after stenting among subgroups A, B, C and D.

The ST segment resolution after stenting	2 h (mv)	4 h (mv)	6 h (mv)	12 h (mv)	24 h (mv)
Group A	3.87 ± 1.96	3.81 ± 1.93	3.93 ± 1.98	4.01 ± 1.92	4.27 ± 1.87
Group B	4.64 ± 2.69	4.67 ± 2.69	4.64 ± 2.69	4.46 ± 2.81	4.55 ± 2.71
Group C	4.91 ± 3.14	5.09 ± 3.21	5.09 ± 3.20	5.00 ± 3.22	5.09 ± 3.21
Group D	3.29±.49	3.09 ± 1.38	3.11 ± 1.58	3.09 ± 1.76	3.18 ± 1.99
*F*	1.11	1.57	1.46	1.19	1.18
*P*	0.358	0.209	0.239	0.323	0.328

**Table 9:** Comparison of TPI after stenting between the experimental group and the control group.

TPI after stenting	12 h (ng/ml)	24 h (ng/ml)
Control group	25.38 ± 8.09	21.01 ± 9.74
Eperimental group	25.08 ± 6.23	22.63 ± 7.93
*T*	0.226	0.45
*P*	0.82	0.76

**Table 10:** Comparison of TPI after stenting in subgroups A, B, C and D.

TPI after stenting	12 h (ng/ml)	24 h (ng/ml)
Group A	25.61 ± 9.38	20.64 ± 9.16
Group B	25.15 ± 9.75	21.38 ± 8.78
Group C	24.84 ± 7.86	22.75 ± 8.28
Group D	25.32 ± 8.25	22.51 ± 9.18
*F*	0.85	0.96
*P*	0.67	0.82

**Table 11:** Comparison of CK-MB after stenting between the experimental group and the control group.

CK-MB after stenting	12 h (U/L)	24 h (U/L)
Control group	247.92 ± 165.52	179.83 ± 96.68
Experimental group	120.27 ± 71.30	93.28 ± 46.61
*T*	2.75	2.45
*P*	0.007	0.016

**Table 12:** Comparison of CK-MB after stenting between subgroups A, B, C, and D.

CK-MB after stenting	12 h (U/L)	24 h (U/L)
Group A	210.27 ± 104.55	104.17 ± 48.23
Group B	285.57 ± 204.64^a,b,c^	136.37 ± 86.48^a,b,c^
Group C	193.20 ± 103.56	96.47 ± 45.51
Group D	166.47 ± 89.01	90.10 ± 48.24
*F*	4.38	3.57
*P*	0.006	0.016

Subgroups were analyzed by LSD method *post hoc* test.

^a^ Means group B compared with group A *P* < 0.05.

^b^ Means group B compared with group C *P* < 0.05.

^c^ Means group B compared with group D *P* < 0.05.

**Table 13:** Comparison of the incidence of no-reflow and slow blood flow between the experimental group and the control group.

Group	Afterthrombus aspiration		After stenting	
	No reflow(%)	Slow blood flow(%)	No reflow(%)	Slow blood flow(%)
Control group	6.7	20	5	16.7
Experimental group	0	6.7	1.7	1.7
*U*	1680	1560	1740	1530
*P*	0.04	0.03	0.31	0.005

**Table 14:** Comparison of the incidences of no-reflow and slow biood flow among subgroups A, B, C and D.

Group	After thrombus aspiration		After stenting	
	No reflow(%)	Slow blood flow(%)	No reflow(%)	Slow blood flow(%)
Group A	6.7	16.7	0	13.3
Group B	6.7	23.3	10	20.0^a,b^
Group C	0	6.7	3.3	3.3
Group D	0	6.7	0	0
*H*	4.1	5.15	6.16	9.03
*P*	0.25	0.16	0.10	0.029

The results of pairwise comparison of subgroups.

^a^ Indicates the *P* < 0.05 of group B compared with group C.

^b^ Indicates the *P* < 0.05 of group B compared with group D.

**Table 15:** Comparison of intraoperative medication between the control group and the experimental group.

Intraoperative medication	Tirofiban		Nitroprusside	
	(%)	Dosage(ml)	(%)	Dosage(ug)
Control group	72.2	5.56 ± 5.16	72.2	355.56 ± 339.93
Experimental group	25	2.50 ± 5.00	75	150.00 ± 100.00
*U*	1890		630	
*T*		1.16		3.87
*P*	0.002	0.97	0.95	0.01

**Table 16:** Comparison of NT-proBNP between control and experimental groups.

NT-proBNP after stenting	24 h (ng/ml)	30 days (ng/ml)	180 days (ng/ml)
Control group	1871.17 ± 1788.70	557.28 ± 680.70	358.65 ± 56.35
Experimental group	1451.91 ± 1405.09	263.28 ± 208.70	186.35 ± 35.72
*T*	1.428	3.20	2.95
*P*	0.156	0.002	0.04

**Table 17:** Comparison of NT-proBNP between subgroups A, B, C and D.

NT-proBNP after stenting	24 h (ng/ml)	30 days (ng/ml)	180 days (ng/ml)
Group A	1450.43 ± 1277.05	524.93 ± 579.82^e,f^	343.45 ± 235.65
Group B	2291.90 ± 2124.16^a,b^	589.63 ± 777.38^c,d^	432.27 ± 176.45^c,d^
Group C	1609.23 ± 1749.43	256.87 ± 212.12	208.37 ± 96.52
Group D	1294.60 ± 951.28	269.69 ± 208.66	167.86 ± 105.42
*F*	2.292	3.445	2.352
*P*	0.042	0.019	0.045

Subgroups were tested by LSD method *post hoc*.

^a^ Means group B compared with group A *P* < 0.05.

^b^ Means group B compared with group D *P* < 0.05.

^c^ Represents group B compared with group C *P* < 0.05.

^d^ Represents group B compared with group D *P* < 0.05.

^e^ Represents group A compared with group C *P* < 0.05.

^f^ Represents group A compared with group D *P* < 0.05.

**Table 18:** Comparison of LVEF between control and experimental groups.

LVEF after stenting	24 h (%)	30 days (%)	180 days (%)
Control group	50.33 ± 6.08	54.51 ± 6.02	55.75 ± 5.66
Experimental group	53.12 ± 5.65	56.62 ± 5.30	57.32 ± 4.55
*T*	2.60	2.04	1.67
*P*	0.01	0.04	0.09

**Table 19:** Comparison of LVEF between subgroups A, B, C and D.

LVEF after stenting	24 h (%)	30 days (%)	180 days (%)
Group A	49.80 ± 5.87^a^	54.03 ± 6.02	55.54 ± 6.24
Group B	50.86 ± 6.34	54.99 ± 6.09	55.97 ± 5.10
Group C	53.42 ± 5.66	56.54 ± 5.20	57.40 ± 4.59
Group D	52.82 ± 5.73	56.70 ± 5.49	57.23 ± 4.58
*F*	2.442	1.517	0.954
*P*	0.068	0.214	0.417

The subgroup was tested by LSD.

^a^ Means *P* < 0.05 between group A and C.

**Table 20:** Stepwise regression analysis of LVEF.

Relevant variables		*β*	Standard error	*P*
LVEF at 24 h after stenting	Location of the lesion	2.27	0.53	0.001
LVEF at 30 days after stenting	Hypertension	2.15	1.13	0.01
	Smoking	2.64	1.27	0.04
	Location of the lesion	2.75	0.32	0.01
LVEF at 180 days after stenting	SO_2_B	2.01	0.40	0.013
	Hypertension	2.03	0.94	0.032
	Smoking	2.93	0.93	0.002
	BMI	1.83	0.84	0.02

**Table 21:** Effects of different suction times on myocardial perfusion and cardiac function prognosis.

Number of suction	2–3	4–5	6–7	≥8
cTFC after thrombus aspiration	30.86 ± 14.8	22.46 ± 11.43	44.94 ± 19.8	25.30 ± 7.1
cTFC after stenting	17.10 ± 5.4	13.81 ± 6.44	18.47 ± 7.14	15.2 ± 5.4
CKMB at 12 h after stenting	207.8 ± 79.3	153.8 ± 108.5	179.4 ± 83.3.3	183.5 ± 104.6
CKMB at 24 h after stenting	98.1 ± 28.7	79.1 ± 47.6	94.8 ± 53.4	89.2 ± 45.1
NT-proBNP at 24 h after stenting	1674.0 ± 1117.4	1222.9 ± 1837.9	1478.9 ± 967.6	1405.9 ± 990.3
NT-proBNP at 30 days after stenting	358.4 ± 256.2	215.5 ± 183.8	310.4 ± 257.9	240.8 ± 106.6
LVEF at 30 days after stenting	54.0 ± 7.0	57.1 ± 7.0	55.4 ± 5.2	55.6 ± 6.1
LVEF at 180 days after stenting	53.6 ± 5.5	57.9 ± 4.4	54.9 ± 4.6	56.4 ± 4.6

## Discussion

The preferred reperfusion strategy for STEMI is primary PCI. Coronary microcirculation dysfunction (CMD), such as slow flow and no-reflow, occurs in 5%–50% of patients when IRA is opened, which aggravate myocardial injury and increase the incidence of heart failure and mortality (6, 7). Relevant literature points out that the mechanism of CMD is related to a variety of factors, such as microcirculation embolism and spasm, ischemia/reperfusion injury, lack of ischemic preconditioning, and individual differences (8). During the interventional procedure for STEMI patients, thrombus and plaque on the vessel wall form tiny thrombus and plaque fragments under the mechanical action of catheters, guidewires, balloons and stents, which lead to embolism and spasm with the blood flow to the distal microcirculation (9). The degree of embolism and spasm is closely related to the accumulation of debris falling off during the operation. A decrease in myocardial perfusion is induced when coronary microcirculation is obstructed by more than 50%. Standardized surgical procedures can effectively reduce iatrogenic microcirculation embolism and spasm.

The treatment of CMD mainly includes drug therapy and non-drug therapy. Thrombus aspiration, as one of the non-drug treatment strategies, can effectively reduce the thrombus burden, reduce the incidence of CMD and improve myocardial perfusion. Meta-analysis studies have shown that patients with high thrombus burden can benefit from thrombus aspiration (10). In the subgroup analysis of the TOTAL study, the thrombus burden after thrombus aspiration in the direct thrombus aspiration group was compared with that after balloon dilation in the PCI group, and it was found that there was no significant difference between the two groups. It is believed that thrombus aspiration and balloon dilation can achieve the same effect of reducing thrombus burden (11). However, thrombus aspiration is to pull the thrombus out of the body to reduce the thrombus load. After balloon dilatation, some thrombus and plaque fragments fall off to the distal end of the blood vessel, which is bound to affect the microcirculation. In this study, optimized thrombus aspiration (experimental group) was performed in patients with TIMI0 blood flow and high thrombus burden, compared with the thrombus aspiration group after balloon dilation (control group). The results showed that the amount of thrombus aspirated in the experimental group was significantly more than that in the control group, and the thrombus load was reduced. In the control group, part of the thrombus fell off to the distal end of the blood vessel after balloon dilatation, and this part of the thrombus would cause obstacles to the vascular microcirculation. In this study, the incidence of slow flow and no-reflow in the experimental group was significantly lower than that in the control group, and the TIMI flow and cTFC after thrombus aspiration in the experimental group were better than those in the control group. Although there was no significant difference in TIMI flow grade after stent implantation between the two groups, the cTFC after thrombus aspiration and stent implantation in the experimental group was significantly lower than that in the control group, indicating that optimized thrombus aspiration can improve myocardial perfusion in patients with high thrombus burden and reduce the incidence of slow flow and no-reflow.

GPIIb/IIIa receptor antagonist and sodium nitroprusside can effectively prevent and improve the incidence of slow flow and no-reflow during PCI (12–15).In this study, the frequency of tirofiban and total dosage of sodium nitroprusside used in the control group were significantly higher than those in the experimental group. The incidence of slow flow and no-reflow in the control group was higher than that in the experimental group, indicating that the optimized thrombus aspiration can affect the immediate blood flow and reduce the incidence of slow flow and no-reflow. TIMI flow grade only represents epicardial blood perfusion and does not represent normal myocardial perfusion (16). This study showed that there was no significant difference in TIMI flow grade between the two groups after stent implantation, but the cTFC of the experimental group was significantly lower than that of the control group, indicating that optimized thrombus aspiration not only affected the immediate coronary flow, but also significantly improved the immediate myocardial perfusion. In TOTAL study, although TIMI flow grade 3 was achieved in both the thrombectomy group and the primary PCI group, microcirculatory disturbances such as cTFC or myocardial staining were not compared. In this study, even if the same TIMI flow grade 3 is achieved after stent implantation, optimized thrombus aspiration can better improve microcirculation and myocardial perfusion.

Related studies have shown that patients with low levels of cTFC have better cardiac function recovery after direct PCI and PTCA in IRA-related vessels (17). The results of this study showed that the CK-MB values of the experimental group at 12 h and 24 h after operation were significantly lower than those of the control group, indicating that the amount of myocardial necrosis in the experimental group was less than that in the control group. Compared with the control group, the optimized thrombus aspiration reduced the amount of myocardial necrosis and played a role in myocardial protection. The cTFC of the experimental group was significantly lower than that of the control group. The LVEF and NT-proBNP of the experimental group were significantly better than those of the control group at 24 h and 30 days after operation, and lasted for 180 days. It is suggested that the first optimization of thrombus aspiration is beneficial to the improvement of cardiac function in the short and medium term.

This study found that LVEF was significantly correlated with total ischemic time after myocardial infarction, smoking, hypertension, BMI and lesion location. Patients with long total ischemic time, smoking, hypertension, obesity and LAD lesions had lower left ventricular ejection fraction. Therefore, patients are advised to quit smoking, strictly control hypertension, lose weight and open the IRA as soon as possible after myocardial infarction to shorten the total ischemic time.

In order to ensure TIMI flow grade 3 after stenting, the amplitude of ST-segment resolution of electrocardiogram at 2 h, 4 h, 6 h, 12 h and 24 h after stenting was observed. There was no significant difference between the two groups, indicating that the electrical changes of necrotic myocardium may be later than myocardial perfusion.

The number of suctioning was stratified into 4 levels:2–3times,4–5times,6–7times and greater than or equal to 8 times. The effects of different levels of suctioning times on TIMI frame count, CK-MB, NT-ProBNP and LVEF were analyzed, concluding that the optimal number of suction was 4–5 times.

In this study, 120 patients were followed up for 6–21 months, and there were no stroke events. The follow-up results showed that there were no significant differences in the cumulative incidence of bleeding events, stroke and MACE events between the two groups. The thrombectomy procedure was standardized by experienced surgeons.

## Data Availability

The original contributions presented in the study are included in the article/**Supplementary Material**, further inquiries can be directed to the corresponding author.
