# Supplementary figures and images for: Effect of optimized thrombus aspiration on myocardial perfusion and prognosis in acute ST-segment elevation myocardial infarction patients with primary percutaneous coronary intervention
Source: Front Cardiovasc Med. 2023 Oct 4;10:1249924. doi: 10.3389/fcvm.2023.1249924 (PMC10584146; doi:10.3389/fcvm.2023.1249924)

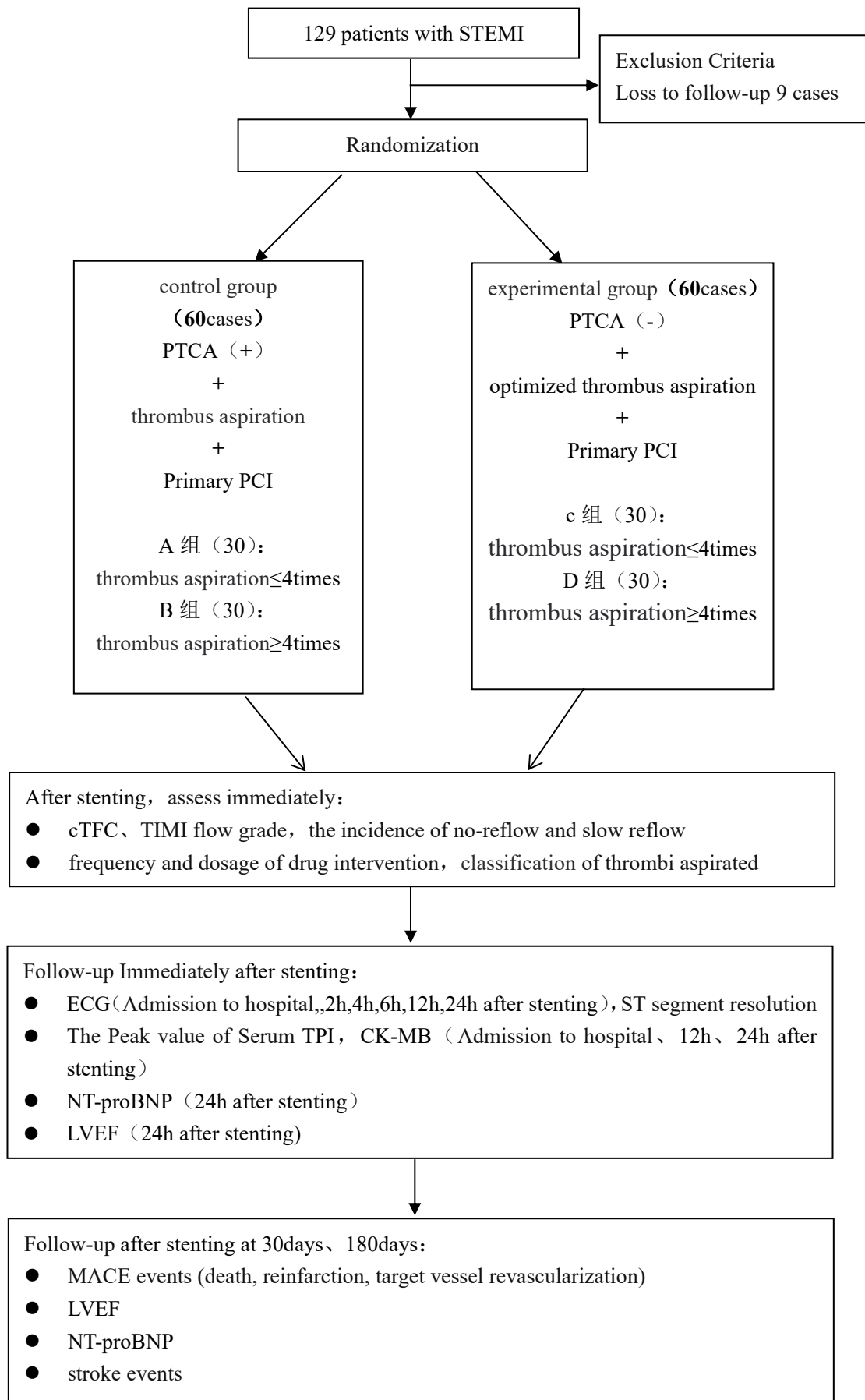

Attached Figure 1: Experimental Design Process

Supplement: Supplementary file 1 [file Datasheet1.pdf]
